# Polymorphism in peptide self-assembly visualized

**DOI:** 10.1073/pnas.2123197119

**Published:** 2022-02-01

**Authors:** Matthew Tirrell

**Affiliations:** ^a^Pritzker School of Molecular Engineering, University of Chicago, Chicago, IL 60637

The spontaneous formation of well-defined supramolecular structures, based on precisely located, finely balanced, and weak, relative to kT, interactions among the assembling subunits, is a useful definition of self-assembly. The recognition that proteins could spontaneously refold from their denatured states (1), demonstrating that the information required to assemble the tertiary structure of a protein is built into the primary structure, was an early indicator of the biological importance and power of self-assembly processes. Spontaneous thermodynamically means moving down a free energy gradient seeking a global minimum, but, of course, it does not mean instantaneous dynamically. Self-assembly processes have pathways, kinetics, and other intricacies of moving over and sampling complex free energy landscapes, and do not always land in the global free energy minimum, demonstrated, for example, by the pathologies related to protein misfolding (2). Organization of native and unnatural proteins and peptides into micelles, sheets, tubules, fibers, filaments, and other multimolecular structures by self-assembly, subject to similar influences as folding of individual protein molecules, also happens in nature and via synthetic molecular engineering. The supramolecular assembly of peptides, often with engineered hydrophobicity, chirality, or other modifications, has proven useful in making materials (3), vaccines (4), and therapeutics, exemplified in the PNAS paper by Pieri et al. (5), in which the structure of the assembly formed from the synthetic octapeptide Lanreotide has been determined at high resolution by cryotransmission electron microscopy (cryo-TEM).

Lanreotide is similar to a natural chemical called somatostatin, which is produced in the body by the hypothalamus and functions *inter alia* to modulate the secretion of growth hormone by the pituitary gland. The nanotube assembly created by this synthetic hormone analog, when delivered transdermally, serves to protect it from enzymatic degradation and to release the therapeutic peptide slowly over time. In general, self-assembly of peptides can enhance the properties and performance of their individual peptide constituents in numerous ways. Hydrophobic modification of peptides, such as those derived from extracellular matrix proteins, often leads to networks of extended, entangled, worm- or rod-like micelles displaying cell adhesion or other signaling activities that could not readily be obtained from the peptides alone (6). Peptide self-assembly defends antimicrobial peptides from degradation through the protection of the cleavage sites and reduction of affinity of proteases (7). A de novo designed amphiphilic peptide PEFEFEFEFEFEP has been exploited as a replication template the catalytic activity of which in generating self-synthesizing materials depends on the ability to assemble into fibrils (8). Self-assembly of peptides with chirality or designed chiral sequences provides not only another means of avoiding degradation by enzymes evolved to cleave bonds between L-amino acids but also the possibility to transfer chirality to larger-scale features of the assembly. This could lead to the formation of hierarchically ordered hybrid chiral films, arrays, or other objects with unique properties, including structural color, optical activity, sensing, templating, catalysis, or other chirality-dependent functions (9).

Assembly of peptides into micelles by hydrophobic modification has been shown to induce stable secondary structure conformations in peptides that would be disordered in an unassembled state (10). This effect can be likened to the stabilizing effect on a secondary structural element, such as an α-helix, arising from the constraints imposed by the protein tertiary structure, and has been shown to promote protein-like functions in these micellar assemblies (11).

Lanreotide is an eight-residue peptide, whose sequence is H_2_N-NahCY^D^WKVCTNH_2_, where Nah is D-naphthylalanine, comprising a precise sequence of aromatic, aliphatic, hydrophobic and hydrophilic, and charged residues. An intramolecular disulfide bond constrains this peptide into a β-hairpin that then assembles in water into nanotubes that are hundreds of micrometers long (5). There is an intriguing assembly phenomenon newly observed in the peptide assemblies of Lanreotide (5), namely, conformational polymorphism. As just cited (10), supramolecular assembly has been shown to drive more ordered average peptide conformation, but assembly in a regular manner in the same nanotube of multiple, individually unique conformations of chemically identical peptides has not previously been identified. This finding of conformational polymorphism by Pieri et al. (5) can certainly have important influences on the properties of peptide assemblies discussed in preceding paragraphs.

**Fig. 1.**
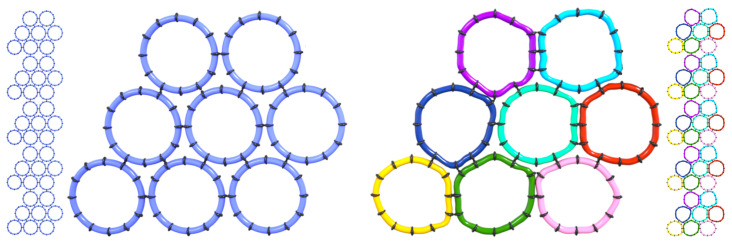
(*Left*) Rigid, regular assemblers organize into a supramolecular structure with all components in identical environments, as indicated by the number of contacts per assembler. (*Right*) Assemblers that can adopt local conformations that increase the number of favorable contacts produce a multisubunit assembling entity.

A structural model of these nanotubes was reported in 2003 (12) based on an array of techniques available at that time, including polarized optical microscopy, low-resolution electron microscopy, X-ray diffraction, and vibrational spectroscopy. A tool is demonstrated in PNAS by Pieri et al. (5), namely, cryoelectron microscopy, that is able reveal the structure of Lanreotide at 2.5-Å resolution. In this work, it is seen that the small Lanreotide peptide adapts to eight slightly different environments via conformational adjustments, which repeat in a highly regular manner over the length of the self-assembled nanotube. Polymorphism of peptide conformation within an assembly of identical components has been inferred, but not directly observed experimentally, from molecular dynamics simulations of a peptide known as MAX1, which is a 20-residue peptide with the sequence VKVKVKVKV^D^PPTKVKVKVKV−NH_2_ (13). Schneider and coworkers (14) have designed this, and a related class of peptides, that undergo triggered folding into facially amphipathic β-hairpins and subsequently self-assemble to form self-supporting hydrogels. Conformational polymorphism has been observed by cryo-TEM in viral capsid proteins (15) and other protein assemblies, where the larger macromolecular size provides more flexibility, but is less expected in assemblies of small peptides. The observation of a repetition of eight slightly different peptide conformations along the nanotube means that this spontaneously formed assembly is formed by joint minimization of the free energy of an ensemble of eight peptide molecules. The ensemble of eight polymorphs becomes the self-assembling entity. A conceptual schematic of this situation is shown in Fig. 1, illustrating the idea that slight local distortions of nearly regular objects can maximize favorable interactions to produce a regular assembly at a larger scale.

The supramolecular assembly of peptides, often with engineered hydrophobicity, chirality, or other modifications, has proven useful in making materials, vaccines, and therapeutics, exemplified in the PNAS paper by Pieri et al., in which the structure of the assembly formed from the synthetic octapeptide Lanreotide has been determined at high resolution by cryotransmission electron microscopy (cryo-TEM).

Cryo-TEM, at resolutions now as small as 1.2 Å (16), has become a game-changing tool not only in protein structure determination but also in protein assemblies, protein ligand–receptor interactions, and, now (5), assemblies of synthetic peptides. The great virtue of microscopy in studying assemblies of peptides and other complexes is that it is the only way to obtain nanoscale structural information whose interpretation is not model dependent (17). In addition to high-resolution cryo-TEM, other innovations that promise, and are beginning to deliver, new structural insights into self-assembled peptides and related materials via real-space imaging include cryoscanning electron microscopy (18), liquid cell EM (19), and superresolution optical microscopy (20), each with its own resolution capabilities and sample preparation requirements. Further developments in the domain of high-resolution visualization can be expected and will be important in understanding peptide self-assembly, and many other significant questions, recalling the Freeman Dyson quotation, “New directions in science are launched by new tools much more often than by new concepts” (21); he gave a more balanced survey subsequently (22).
